# HOXD3 targeted by miR-203a suppresses cell metastasis and angiogenesis through VEGFR in human hepatocellular carcinoma cells

**DOI:** 10.1038/s41598-018-20859-3

**Published:** 2018-02-05

**Authors:** Lumin Wang, Dongdong Tong, Qingqing Guo, Xiaofei Wang, Fei Wu, Qian Li, Juan Yang, Lingyu Zhao, Yannan Qin, Yingxun Liu, Chen Huang

**Affiliations:** 1 Department of cell Biology and Genetics, School of Basic Medical Sciences, Xi’an Jiaotong University Health Science Center, Xi’an, China; 2 Key Laboratory of Environment and Genes Related to Diseases, Xi’an Jiaotong University Health Science Center, Xi’an, Shaanxi China; 3 Cardiovascular Research Center, Xi’an Jiaotong University Health Science Center, Xi’an, Shaanxi P.R. China

## Abstract

Hepatocellular carcinoma (HCC), one of the most common aggressive tumors worldwide has a relatively high mortality rate among malignant tumors. MicroRNAs (miRNAs), acting as tumor suppressors, are involved in the regulation of invasion, metastasis, and angiogenesis of tumor cells. However, a potential role for miR-203a in HCC has not been described yet. In this study, we show that miR-203a markedly suppresses HCC cell migration, invasion, and angiogenesis. In addition, the transcription factor HOXD3 appears to be a direct target of miR-203a. HOXD3 knockdown substantially decreased HCC cell migration, invasion, and angiogenesis, effects similar to those seen for miR-203a expression. Rescuing the function of HOXD3 attenuated the effect of miR-203a overexpression in HCC cells. Furthermore, HOXD3 can directly target the promoter region of VEGFR and increase VEGFR expression. Taken together, our findings indicate that miR-203a inhibits HCC cell invasion, metastasis, and angiogenesis by negatively targeting HOXD3 and suppressing cell signaling through the VEGFR pathway, suggesting that miR-203a might represent a potential therapeutic target for HCC intervention.

## Introduction

Hepatocellular carcinoma (HCC) is as a malignant tumor of the digestive system and is the third leading cause of cancer-related mortality worldwide^1,2^. Owing to the lack of specific early symptoms or effective diagnosis and tumor biomarkers, the survival rate for HCC is extremely low. Thus, it is necessary to identify novel and efficient biomarkers that can be used for diagnosis, and act as therapeutic targets, in human HCC. Several studies have indicated that deregulation or dysfunction of miRNAs may contribute to the development of cancer^3,4^.

MicroRNAs (miRNA) are a group of small noncoding RNAs that play an essential role in cancer development by regulating the activities of specific mRNA targets^5^. It is well known that miRNAs, acting as either oncogenes or tumor suppressors, participate in numerous biological processes, such as invasion, metastasis and angiogenesis^6–9^. Similar to other members of the miR-203 family, miR-203a has been reported to act as an anti-oncogenic miRNA in some cancers^10,11^. However, its role in HCC metastasis has not been described yet.

Recent reports have demonstrated that several genes or signaling pathways, including E2F3, MET, and the PTEN/AKT signaling pathway, may be involved in HCC metastasis and angiogenesis^12–14^. The genes of HOX family are conserved transcription factors that determine cellular identity during development. Numerous studies have shown that dysregulated HOX expression plays a regulatory role in tumor metastasis and angiogenesis^15–17^. HOXD3 is the third paralog of the HOXD gene family, and plays a pivotal role in cancer cell invasion, metastasis, and angiogenesis. Previous studies have shown that overexpression of HOXD3 contributes to an increase in extracellular matrix adhesiveness and enhances the expression of β3 integrin in A549 cells and erythro-leukemia HEL cells^18,19^. In our previous study, we found that miR-203a targets *HOXD3* and, through the EGFR/AKT and ERK signaling pathways, leads to suppression of HCC cell proliferation^20^. However, the underlying molecular mechanisms by which miR-203a regulates invasion, metastasis, and angiogenesis in HCC, via targeting of *HOXD3* in HCC cells, has yet to be fully elucidated. Furthermore, as HOXD3 is a member of a transcription factor family that contains homeodomains, it can bind to the promoter region of numerous target genes and regulate their expression. However, the mechanism by which HOXD3 regulates the expression of oncogenes and tumor suppressors in tumor proliferation, invasion, metastasis, and angiogenesis has not been reported. In earlier studies, we found that HOXD3 targets the promoter region of *EGFR* and regulates the expression of EGFR as well as its downstream proteins^20^.

In this study, by overexpressing or silencing miR-203a and HOXD3 expression in HCC cells, we show that *HOXD3* can be targeted by miR-203a and directly regulates the expression of VEGFR to inhibit HCC metastasis, invasion, and angiogenesis. The present study therefore suggests that miR-203a may act as a tumor suppressor and HOXD3 may play the role of an oncogene; and thus, may provide a beneficial strategy for future HCC therapy.

## Materials and Methods

Both tumor and non-tumor tissues were histologically confirmed. Informed consent was obtained from each patient and was approved by the Institute Research Ethics Committee at Cancer Center, Xi’an Jiaotong University. In addition, all experimental protocols were performed under the guidelines of the Xi’an Jiaotong University Health Science Center and approved by the Institute Research Ethics Committee at Cancer Center, Xi’an Jiaotong University.

### Cell culture and HCC tissues

SMMC-7721 and Hep3B cells were cultured in in RPMI 1640 containing 10% fetal bovine serum (FBS) at 37 °C and in 5% CO2. All reagents used for cell culture media were from PAA Laboratories GmbH. 48 HCC and normal tissues were collected from the Pathology Department of the Second Affiliated Hospital (Xi’an Jiaotong University, Xi’an, China). No local or systemic treatment had been conducted before operation.

### RNA extraction, retrotranscription and quantitative real-time PCR(qRT-PCR)

For HCC tissues the total RNA was extracted using the RecoverAll TM Total Nucleic Acid Isolation Kit (Ambion, Austin, TX, USA) according to the manufacturer’s protocol. qRT-PCR was performed according to the methods described previously^11^.

### Plasmids construction and transfection

The construction of miR-203a and HOXD3 expression vectors and the synthesis of ASO-miR-203a (antisense oligonucleotide of miR-203a, miR-203a inhibitor), si-ctrl and si-HOXD3 were performed as described previously^20^. Transfections were carried out using Lipofectamine-2000 (Invitrogen, Carlsbad, CA) according to the manufacturer’s instructions.

### Cell invasion assay

The Transwell chambers (Millipore, Billerica, MA, USA) (8-µm pore size) were coated with Matrigel (BD Biosciences, Franklin Lakes, NJ, USA) (15 µg/filter). Cells (1.0 × 10^4^) in serum-free medium were plated into the upper chamber and the bottom wells were filled with complete medium. The cells were incubated at 37 °C for 48 h, and then cells in upper chamber were removed using cotton swabs. Cells invading the bottom of the membrane were stained with 1% crystal violet. Quantitative analysis of invasion rates was performed by solubilization of crystal violet and obtaining spectrophotometric readings at OD 490 nm. Five random fields from each membrane were photographed and counted for statistical analysis.

### Wound-healing assay

Cells were seeded in 6-well culture plates at a density of 4 × 10^5^ cells/2 mL/well. Once the cells had grown to 80% confluence, a single scratch wound was generated with a 200-µl disposable pipette tip. The extent of wound closure was measured 48 h after wounding.

### Western blot analysis

Total cell lysates from different experiments were obtained by lysing the cells in RIPA buffer. Protein concentration was calculated with a Pierce BCA protein assay kit (Thermo Scientific, Rockford, IL, USA). Protein was then separated with an 8–10% SDS-PAGE (Invitrogen) gel; transferred to a nitrocellulose membrane; and incubated with the VEGFR, E-cadherin, N-cadherin, MMP9 and β-actin antibodies (Cell Signaling Technology; diluted 1/500). After the membrane was washed three times with TBST, it was incubated with a goat anti-rabbit antibody (Bioworld; diluted 1/5000). Relative protein expression was then normalized to β-actin levels in each sample.

### *In vitro* HUVEC tube network formation assay

For the tube network formation assay, each well of a 96-well plate was pre-coated with 50 μl of Matrigel (BD, USA) and allowed to polymerize for 30 min at 37 °C. Next, miR-ctrl, miR-203a, ASO-NC, ASO-miR-203a, si-ctrl, siHOXD3, HOXD3-ctrl and HOXD3 were transfected into HUVECs for 24 h, incubated them without serum free medium for 24 h, and transfectant HUVECs (4 × 10^4^) were resuspended in 100 μl of conditioned media with 1% FBS and seeded on Matrigel-coated 96-well plate. HUVECs were incubated for 8 h to allow formation of tube-like structures. Number of formed tube were counted and compared from three different fields under a light microscope.

### Chromatin immunoprecipitation assay (ChIP)

The binding of HOXD3 to the promoter of VEGFR was tested using ChIP analysis. CHIP was performed according to the methods described previously^20^.

### Statistical analysis

Each experiment was repeated at least three times. Data are presented as mean ± SD. Unless indicated, the statistical significance of differences between the two groups was analyzed using a Student’s t-test (two-tailed). All statistical analyses were performed using the SPSS13.0 software (SPSS, Chicago, IL, USA).

## Results

### miR-203a suppresses HCC invasion and angiogenesis both *in vitro* and *in vivo*

**Figure 1:**
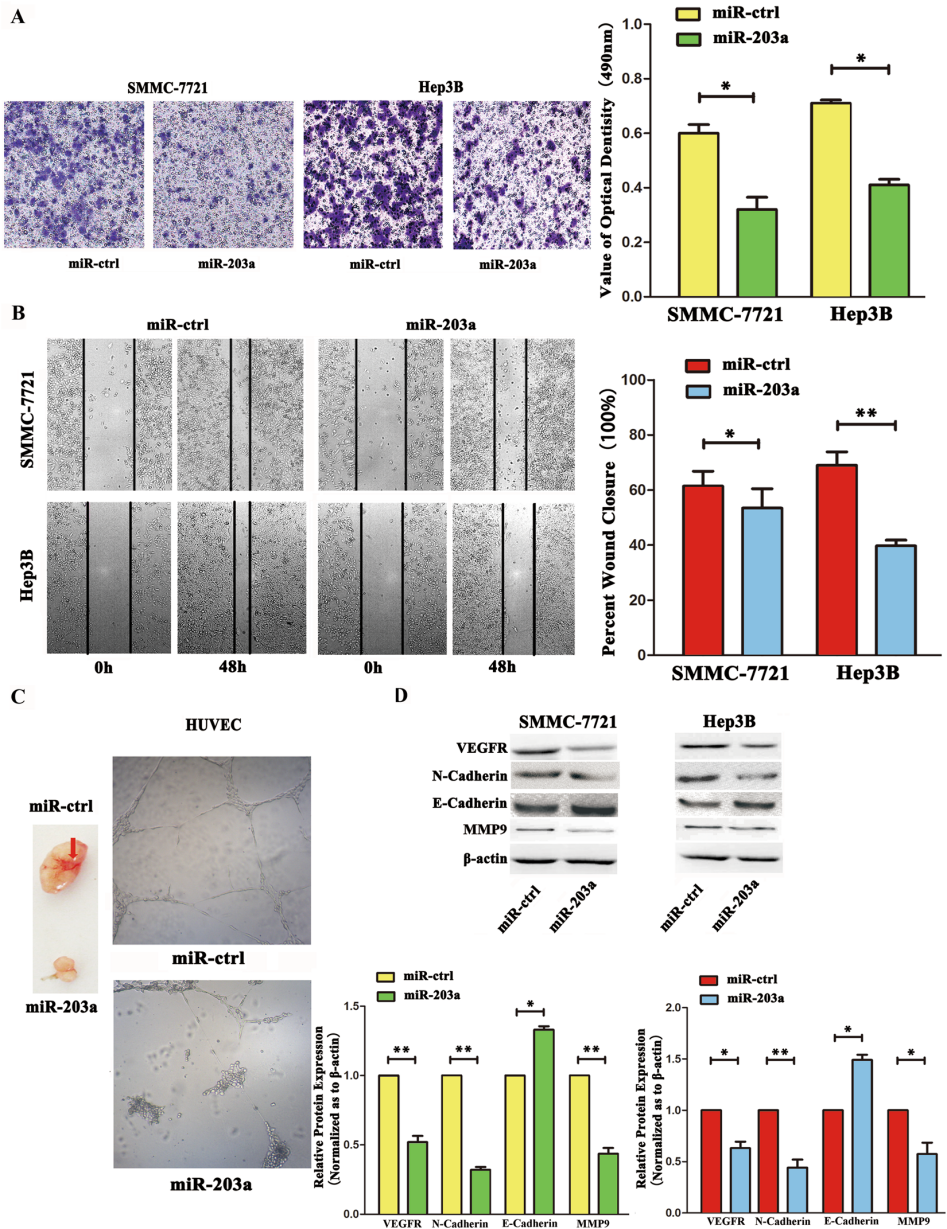
Overexpression of miR-203a suppressed HCCs metastasis, invasion and angiogenesis. **(A)** Transwell analysis of SMMC-7721 and Hep3B cells after transfected with miR-203a and its ctrl, quantitative analysis of the invasion rates by solubilization of crystal violet and spectrophotometric reading at OD 490 nm. **(B)** Wound-healing assays of SMMC-7721 cells and Hep3B after treatment with miR-ctrl, miR-203a. The relative wound closure (100%) represents the metastasis capacity of SMMC-7721 and Hep3B cells. **(C** Left) Tube formation assay in HUVECs after transfected with miR-ctrl and miR-203a *in vitro*. (**C** Right) Angiogenesis assay in SMMC-7721 after transfected with miR-203a and its ctrl. **(D)** Effects of miR-203a overexpression on protein expression of EMT markers and related proteins by western blot. β-actin was detected as an internal control (*P < 0.05; **P < 0.01, Student’s t test).

To investigate the function of miR-203a in HCC invasion, we applied two methods: a trans-well invasion assay and a wound healing assay. In the trans-well invasion assay, SMMC-7721 and Hep3B cells overexpressing miR-203a showed significantly decreased invasive abilities compared with cells transfected with the vector control (Fig. 1A). In the wound-healing assay, we found that overexpression of miR-203a inhibited the migration of SMMC-7721 and Hep3B cells (Fig. 1B). To determine whether miR-203a also regulates angiogenesis, human umbilical vein endothelial cells (HUVECs) were transfected with either a miR-203a-expressing vector or its control. Notably, miR-203a expression decreased angiogenesis by attenuating tube formation in HUVECs (Fig. 1C). *In vivo*, we used lentiviral vectors to stably express miR-203a in SMMC-7721 cells. LV-miR-203a-infected and LV-CN-infected (control) cells were injected subcutaneously into the opposite posterior flanks of the four nude mouse and angiogenesis was measured after 4 weeks. As shown in Fig. 1C, angiogenesis was significantly suppressed by LV-miR-203a compared with the control. Western blotting showed that miR-203a expression inhibited the expression of VEGFR, MMP9 and N-cadherin while increasing the expression of E-cadherin (Fig. 1D)

### Inhibition of miR-203a expression increases the HCCs invasion and angiogenesis ***in vitro***

#### Inhibition of miR-203a expression increases HCC cell invasion and angiogenesis *in vitro*

**Figure 2:**
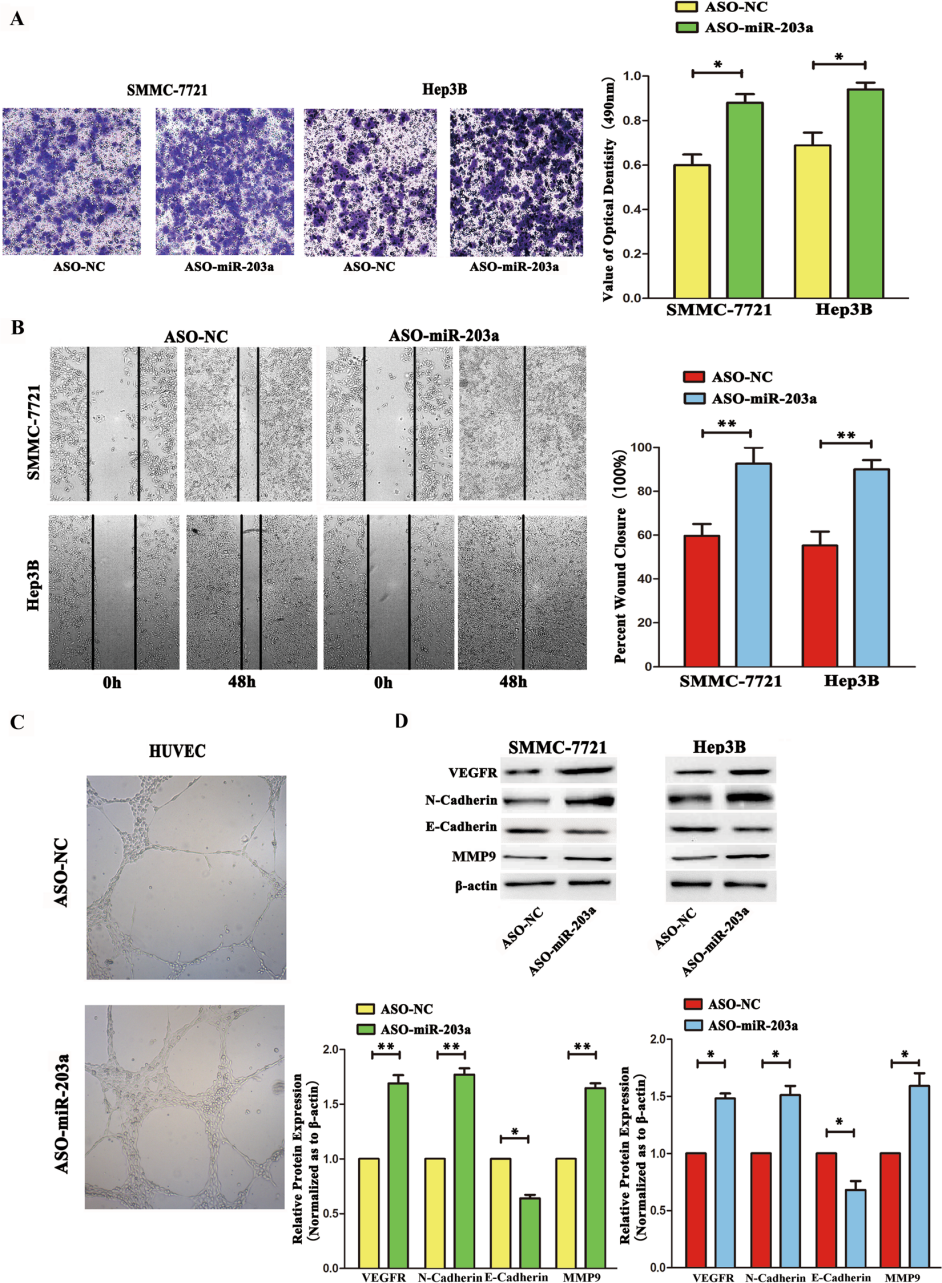
Inhibitor of mir-203a increased HCCs metastasis, invasion and angiogenesis. **(A**) Transwell analysis of SMMC-7721and Hep3B cells after transfected with ASO-NC and ASO-miR-203a. Bottom, quantitative analysis of the invasion rates by solubilization of crystal violet and spectrophotometric reading at OD 490 nm. **(B)** Wound-healing assays with SMMC-7721 cells and Hep3B treated with ASO-miR-203a and its control. The relative wound closure (100%) represents the metastasis capacity of SMMC-7721 and Hep3B cells. **(C)** Tube formation assay in HUVECs transfected with ASO-NC and ASO-miR-203a. **(D)** Effects of miR-203a knockdown on protein expression of EMT markers and related proteins by western blot. β-actin was detected as an internal control (*P < 0.05; **P < 0.01, Student’s t test).

To investigate the function of miR-203a further, the miR-203a inhibitor or its control were transfected into SMMC-7721 and Hep3B cells. Using trans-well invasion and wound healing assays, we found that inhibition of miR-203a expression increased HCC cell invasion and angiogenesis (Fig. 2A,B). The same trend was seen in the tube formation assay, with increased tube formation observed in cells transfected with the miR-203a inhibitor (Fig. 2C). By western blotting, the expression of VEGFR, N-cadherin and MMP9 were found to be up-regulated, whereas that of E-cadherin was down-regulated, in HCC cells transfected with the miR-203a inhibitor (Fig. 2D). These data support the hypothesis that abnormal expression of miR-203a affects invasion, metastasis, and tube formation in HCC cells.

### HOXD3 knockdown has effects similar to those of miR-203a overexpression in HCC cells

**Figure 3:**
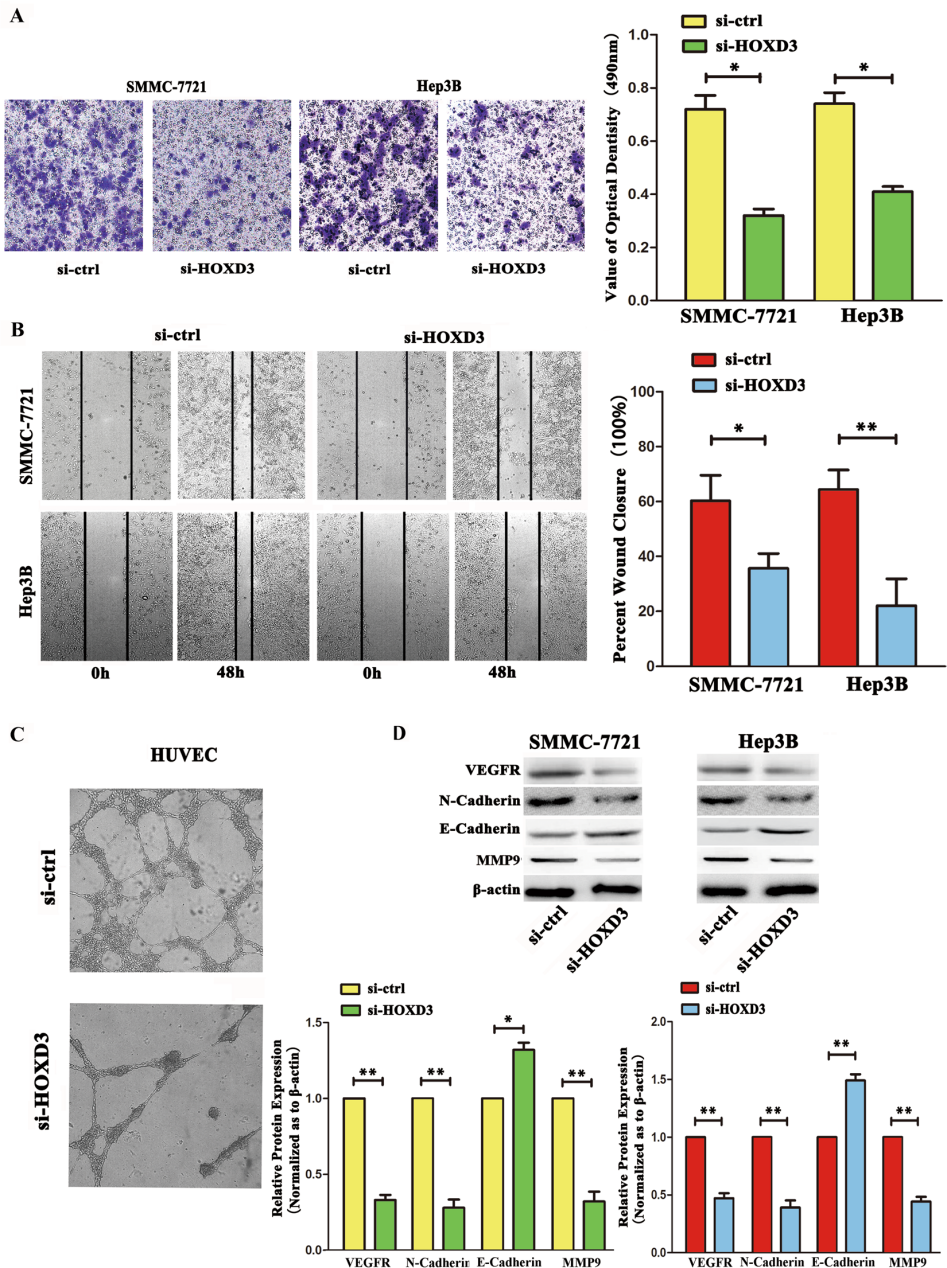
miR-203a inhibits cells invasion and angiogenesis through HOXD3. **(A**) The invasion viability of SMMC-7721 cells and Hep3B was determined using the Transwell invasion assay, quantitative analysis of the invasion rates by solubilization of crystal violet and spectrophotometric reading at OD 490 nm. **(B)** Wound-healing assays of HCCs after treatment with si-ctrl and si-HOXD3. Representative images were captured at 0 and 48 h after transfection of si-ctrl and si-HOXD3. **(C)** Angiogenesis assay in HUVECs transfected with si-HOXD3 and its control. **(D)** The expression of VEGFR and downstream genes E-cadherin N-cadherin, MMP9 and by using western blot. β-actin as an internal control was detect (**P < 0.01, Student’s t test).

In a previous study, we confirmed that miR-203a could target the 3’-UTR region of the *HOXD3* mRNA. In this study, in order to clarify the role of HOXD3 in invasion and angiogenesis of HCC cells, we silenced HOXD3 expression using RNA interference (RNAi). Using the trans-well invasion, wound healing, angiogenesis, and western blot assays, we found that silencing HOXD3 resulted in the suppression of cell invasion, migration, angiogenesis, downregulation of VEGFR, N-cadherin and MMP9 expression, and up-regulation of E-cadherin expression, all of which were comparable to the effects seen following miR-203a overexpression in SMMC-7721/Hep3B cells (Fig. 3).

### Overexpression of HOXD3 attenuates the effects of miR-203a on HCC cells

**Figure 4:**
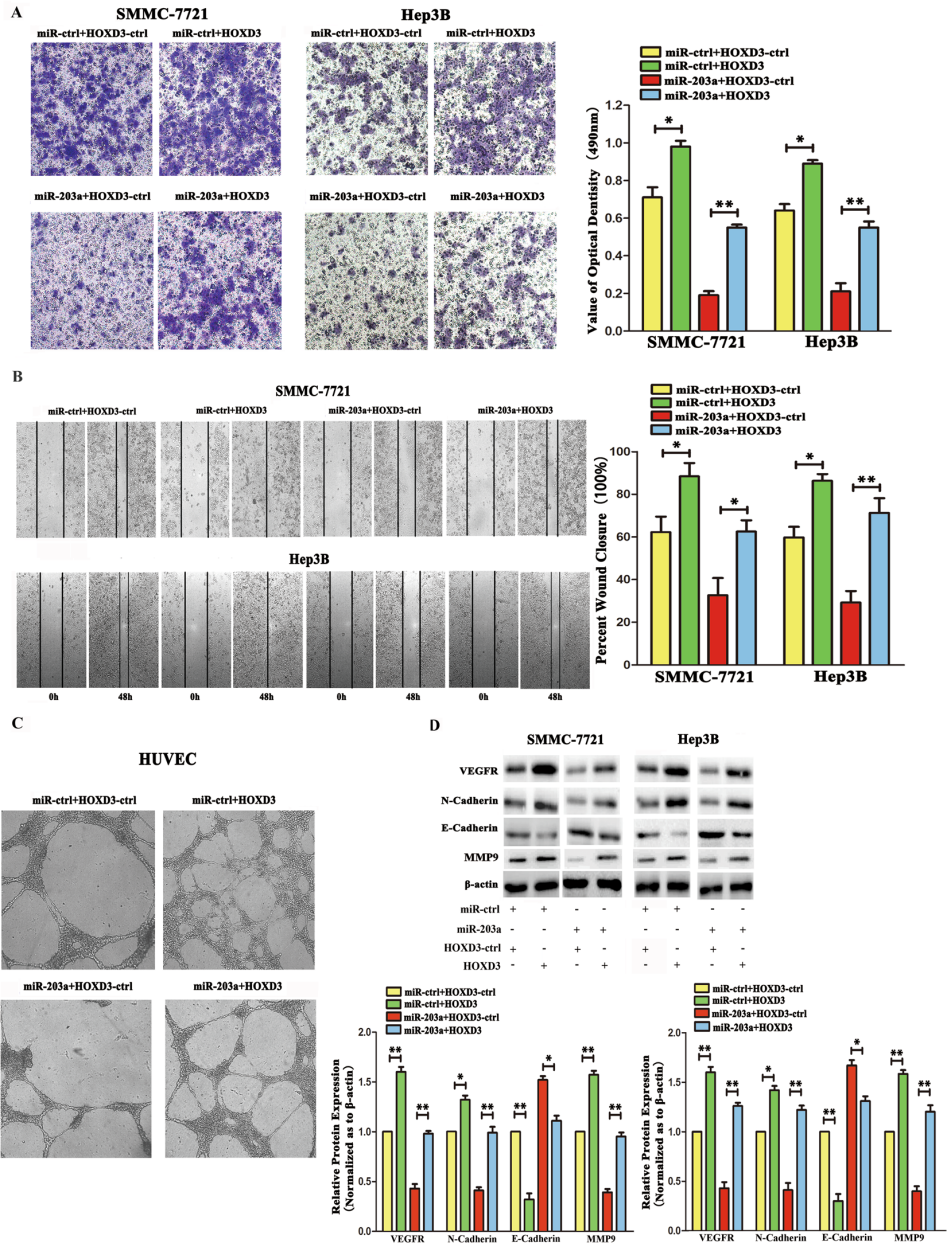
miR-203a rescues HOXD3 induced cellular invasion and angiogenesis in HCCs. **(A**) **S**MMC-7721 and Hep3Bcells invasion was verified by Transwell assay. Quantitative analysis of the invasion rates by solubilization of crystal violet and spectrophotometric reading at OD 490 nm. **(B)** The wound-healing assay was performed to determine the immetastasis of HCCs. (**C**) Tube formation assay in HUVECs transfected with ASO-NC and ASO-miR-203a. **(D)** The expression of VEGFR, EMT marker genes E-cadherin, N-cadherin and MMP9 were assayed by western blot analysis. β-actin was also detected as an internal control (*P < 0.05; **P < 0.01, Student’s t test).

To further demonstrate that miR-203a suppresses migration through HOXD3, we constructed an HOXD3 overexpression vector, which was then co-transfected with either miR-ctrl or miR-203a into SMMC-7721 cells and Hep3B cells. Overexpression of HOXD3 increased the number of invasive cells and angiogenesis compared to that in control transfected cells. However, after co-transfection of miR-203a and HOXD3, we found that the expression of miR-203a attenuated these effects of HOXD3 in SMMC-7721 and Hep3B cells (Fig. 4A–C). The expression of HOXD3 downstream effectors was also evaluated by western blot analysis, following cotransfection with miR-ctrl and HOXD3-ctrl or miR-ctrl and HOXD3 in HCCs. Expression of VEGFR, N-cadherin, MMP9 were up-regulated and E-cadherin was down-regulated after transfection with HOXD3. Meanwhile, compared with cotransfected with miR-203a and HOXD3-ctrl, the expression of VEGFR, MMP9, N-cadherin was upregulated after cotransfected with miR-203a and HOXD3 (Fig. 4D). T These results further suggest that miR-203a suppresses tumor invasion and angiogenesis by directly targeting *HOXD3*.

### HOXD3 activates VEGFR expression

**Figure 5:**
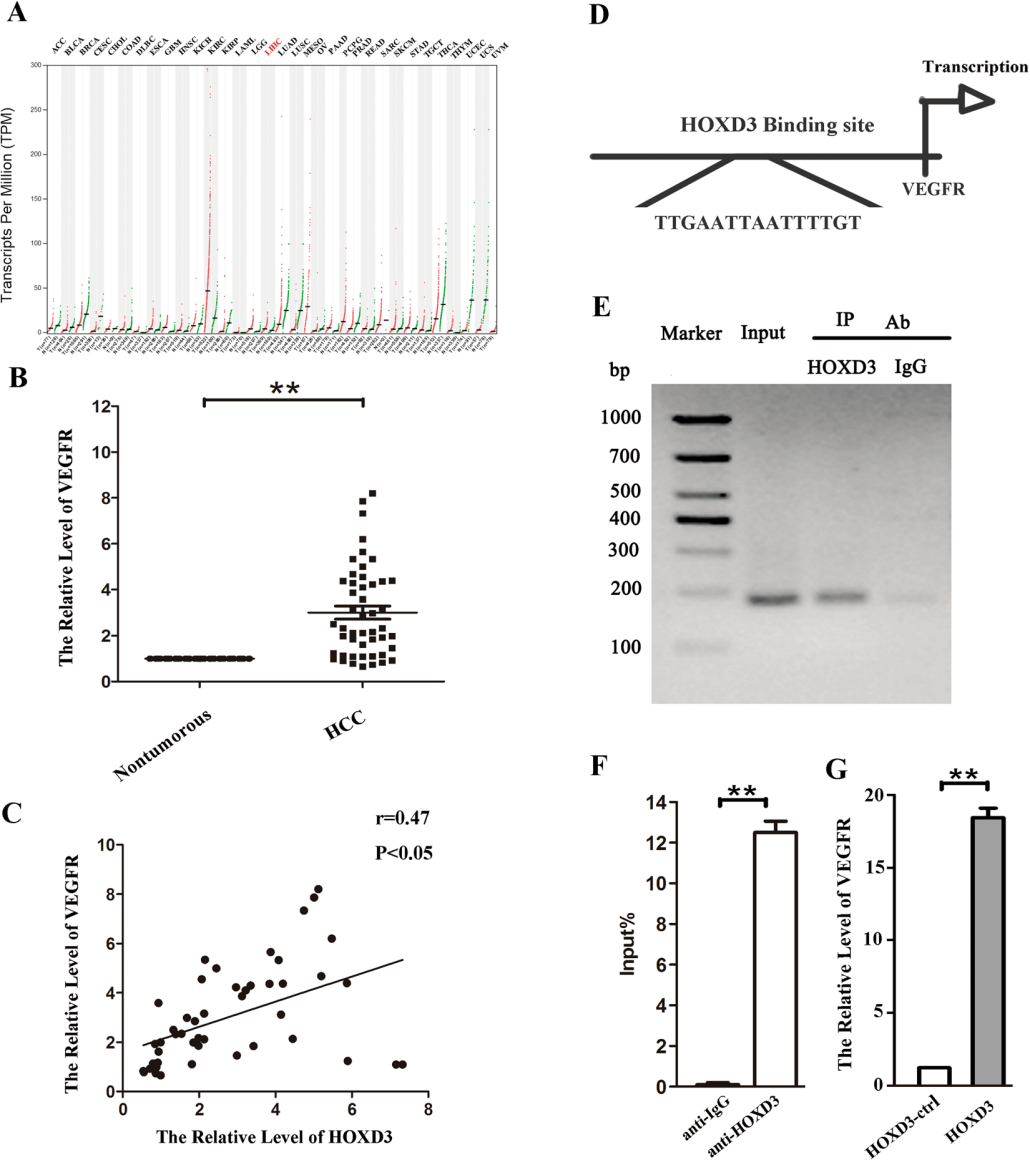
HOXD3 induces VEGFR promoter activity in HCCs. **(A)** The expression of VEGFR in tumor tissues were analyzed by TCGA data base. **(B)** The expression levels of VEGFR mRNA in HCC and healthy tissues were analyzed by qRT-PCR. **(C)** The relationship between HOXD3 and VEGFR were assayed by Pearson’s r. **(D)** Schematic diagram of the putative VEGFR promoter with one potential HOXD3 response element. **(E)** The interaction of HOXD3 with VEGFR was shown using ChIP assays with control (rat IgG) or anti-HOXD3 antibody. **(F)** qRT-PCR analysis was performed with primers spanning predicted HOXD3 of VEGFR. **(G)** The expression of VEGFR was analyzed by qRT-PCR after transfection with HOXD3 vector or empty vector in SMMC-7721 cells (*p < 0.05, **p < 0.01).

Using the TCGA database to analyze the expression of VEGFR in tumor tissues, we confirmed that expression of VEGFR was upregulated in LIHC (liver hepatocellular carcinoma) (Fig. 5A). Next, by qRT-PCR, we confirmed the aberrant expression levels of VEGFR in HCC tissues, compared with those in their respective normal tissues (Fig. 5B). VEGFR levels were also positively correlated with HOXD3 expression (Fig. 5C). To elucidate the relationship between VEGFR and HOXD3 in HCC cell, we used a bioinformatics approach to predict the site in the *VEGFR* gene that is targeted by HOXD3 (Fig. 5D). In further support of this notion, a ChIP analysis revealed that there is a putative HOXD3-binding site located in the upstream region of the *VEGFR* gene (Fig. 5E and F); overexpression of HOXD3 in HCC cells resulted in an increase in VEGFR expression (Fig. 5G), suggesting that HOXD3 is involved in the regulation of VEGFR expression.

## Discussion

HCC is one of the most common aggressive tumors worldwide and has a relatively high mortality rate among malignant tumors^21^. Previous studies have shown that miRNAs participate in a broad range of biological processes, including cell cycle, apoptosis, migration, and invasion^22–24^. In HCC, miRNAs play a crucial role in the important molecular and cellular mechanisms that govern tumorigenesis^25,26^. In our previous and new researches, we found miR-203a was downregulated in HCC tissues (Fig. S1) and HCCs. Combining with the assay of correlation between miR-203a expression levels and clinicopathological characteristics of HCC patients, it showed that miR-203a expression levels were significantly associated with tumor TNM stage (Table 1), suggesting that miR-230a plays a tumor-suppressive role. However, the function of miR-203a in tumor invasion, migration, and angiogenesis was not clear. In our current study, we hypothesized that reduced expression of miR-203a may be responsible for metastasis and angiogenesis in HCC cells.

**Table 1:** Patient characteristics and clinicopathologic correlation of miR-203a and VEGFR expression.

Characteristics	Number of cases	miR-203a expression		P-value	VEGFR expression		P-value
		High	Low		High	Low	
Age (years)				0.821			0.324
≥60	24	7	17		21	3	
<60	34	9	25		25	9	
Gender				0.089			0.716
Male	45	10	35		35	10	
Female	13	6	7		11	2	
Histology				0.060			0.078
Well	20	9	11		15	5	
Moderate	6	2	4		3	3	
poor	32	5	27		28	4	
pTNM Stage				0.046			0.007
I	15	2	13		13	2	
II	14	3	11		7	7	
III	22	6	16		21	1	
IV	7	5	2		5	2	

Our data showed that overexpression of miR-203a inhibits both metastasis and angiogenesis in HCCs, and a miR-203a inhibitor increased metastasis and angiogenesis in HCC cells. Furthermore, from miR-203a gain- and loss-of-function studies, we discovered that overexpression of miR-203a reduced tumor angiogenesis (Figs 1, 2). To verify the role of miR-203a in tumors, the TCGA database was used to analyze the relationship between miR-203a and survival rate. The data showed that HCC patients with high levels of miR-203a expression had high survival rates (Fig. S2).

In our previous research, we found that *HOXD3* is a target gene for miR-203a^20^. The *HOXD3* gene, which is a member of the *HOX* transcription factor gene family, has been shown to be expressed in several tumor cell lines that exhibit enhanced invasion and metastasis through the coordinated expression of metastasis-associated factors. In breast cancer, for example, the expression of HOXD3 has been shown to be closely associated with integrin β3 expression^27^. Furthermore, the overexpression of HOXD3 in A549 cells also elevated the expression levels of uPA and MMP-2 compared to that in parental cells and control transfected cells^18^. In this study, we found that knockdown of *HOXD3* decreased invasion, migration, and angiogenesis of HCC cells (Fig. 3). However, the effect of overexpression of HOXD3 was contrary to lose the function of HOXD3, and overexpression of HOXD3 could eliminate the effects of miR-203a on HCC cells (Fig. 4).

We also demonstrated that aberrant expression of HOXD3 affected the expression of VEGFR. Given that HOXD3 is a transcription factor, we used a bioinformatics tool (http://genome.lbl.gov/, http://ecrbrowser.dcode.org/ and http://jaspar.genereg.net/) to predict the binding sequences for HOXD3, and found that five sequences, upstream to the *VEGFR* gene locus, interacted with HOXD3. Furthermore, we performed a ChIP assay to examine the relationship between HOXD3 and VEGFR, and found that HOXD3 could indeed target an upstream region in the *VEGFR* gene (Fig. 5), suggesting that HOXD3 might be involved in the regulation of VEGFR expression in HCC. Next, we correlated the levels of VEGFR expression and the clinicopathological characteristics of HCC patients, as shown in Table 1. Strikingly, VEGFR expression levels were significantly associated with tumor histology and TNM stage, suggesting that upregulated VEGFR protein expression might be involved in HCC progression.

It is well-known that local invasion and distant metastasis are the leading causes of poor prognosis in patients with HCC. Epithelial-mesenchymal transition (EMT) contributes significantly to progression and metastasis in cancer cells. During EMT, epithelial cells lose the expression of epithelial markers and acquire a mesenchymal phenotype, playing an important role in cancer malignancy, metastasis, and recurrence^28^. The PI3K/AKT oncogenic kinase signaling pathway is often upregulated in cancer initiation and invasion. VEGFR is another molecule that has also been clearly shown to be positively associated with cancer invasion through activating downstream PI3K/AKT signaling and inducing EMT^29^.

**Figure 6:**
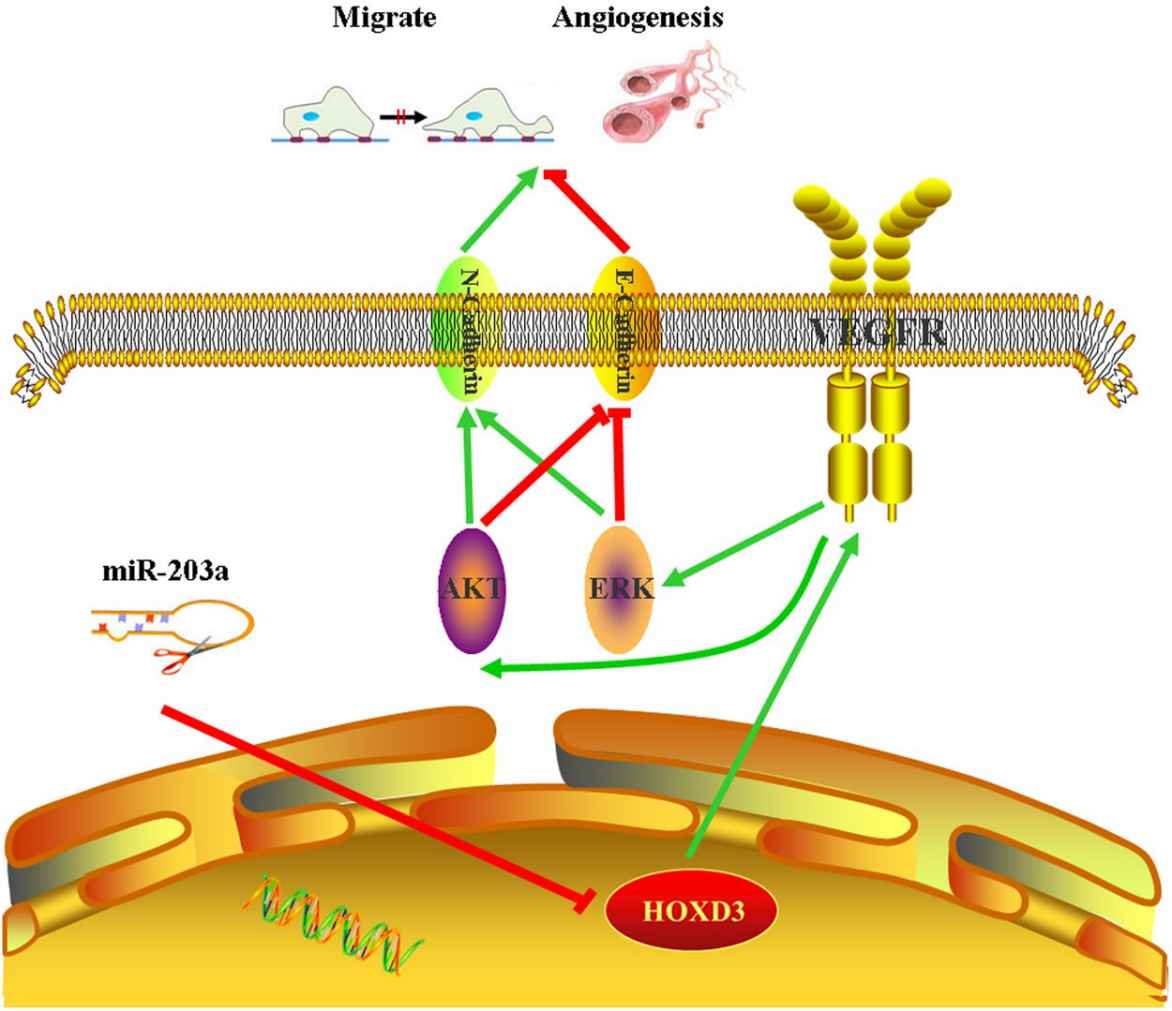
HOXD3 targeted by miR-203a suppresses cell metastasis and angiogenesis directly through the VEGFR signal pathway in human hepatocellular carcinoma cells.

The functions of vascular endothelial growth factor (VEGF), triggered by external stimuli, are initiated through the activation of intracellular signal cascades involving specific kinases^30^. VEGF interacts with its receptors and activates signal transduction pathways, including AKT and ERK, to regulate cell proliferation and migration^31,32^. In a previous study, we found that overexpression of miR-203a or knockdown of *HOXD3*, resulted in the suppression of AKT and ERK phosphorylation and activation. Using a bioinformatics approach (https://string-db.org/, http://genemania.org/), we discovered that HOXD3, VEGFR, AKT, ERK, BCL2, BAX, EGFR, E-Cadherin, N-Cadherin, CCNB1, and CDK1 were all part of one signaling network (Fig. S3). From the current study, we showed that the expression of EMT marker E-cadherin and N-cadherin protein levels has changed, when gain and lose the function of miR-203a and HOXD3. VEGF is a key regular of angiogenesis that promotes survival and induces the proliferation and migration of endothelial cells (ECs), thereby contributing to the formation of new blood vessels^33^. We used HUVEC cells to assess the function of miR-203a in tumor angiogenesis *in vitro*, and xenograft tumor growth to demonstrate the role of miR-203a in angiogenesis *in vivo*. Based on these studies, we propose that miR-203a targets *HOXD3* directly and acts through the VEGFR signaling pathway to suppress angiogenesis (Fig. 6).

In this context, our results demonstrate that overexpression of miR-203a inhibits *HOXD3*, which then indirectly leads to down-regulation of the VEGFR pathway, thereby suppressing tumor invasion, migration, and angiogenesis in HCC.

## Electronic supplementary material

Supplementary information
