# Supplementary material for: HOXD3 targeted by miR-203a suppresses cell metastasis and angiogenesis through VEGFR in human hepatocellular carcinoma cells
Source: Sci Rep. 2018 Feb 5;8:2431. doi: 10.1038/s41598-018-20859-3 (PMC5799159; doi:10.1038/s41598-018-20859-3)

HOXD3 targeted by miR-203a suppresses cell metastasis and angiogenesis through the VEGFR in human hepatocellular carcinoma cells

Lumin Wang[1], Dongdong Tong[1], Qingqing Guo[2], Xiaofei Wang[2], Fei Wu[1], Qian Li[1], Juan Yang[1], Lingyu Zhao[1], Yannan Qing[1] and Yingxun Liu[1] and Chen Huang[1,2,3*].

Supplementary Information

Supplementary Figure 1.The expression of miR-203a was reduced in clinical sepecimens. The average expression level of miR-203a in HCC (n=10) and normal tissues (n=10). The expression ofmiR-203a was normalized to U6 RNA.

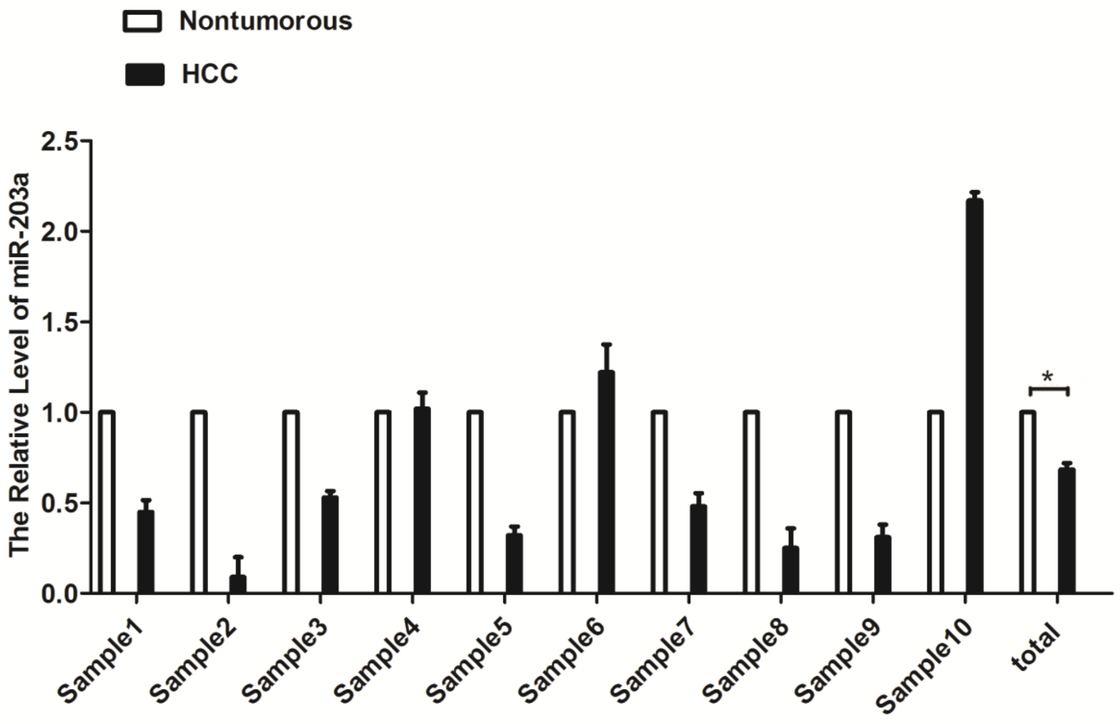

Supplementary Figure 2. The relationship between miR-203a and survival rates. (**A**) The relationship between the expression of miR-203a and overall survival. (**B**) the relationship between the expression levels of miR-203a and disease free survival.

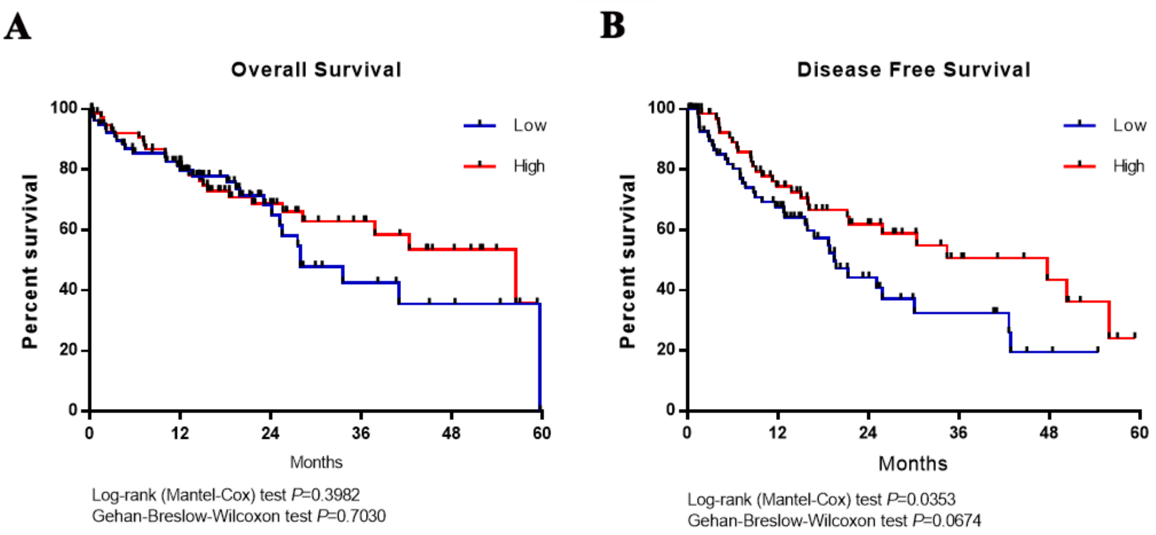

Supplementary Figure 3. VEGFR related signal transduction pathway. (**A**) and (**B**) The assay of bioinformatics showed that HOXD3, VEGFR, AKT, ERK, BCL2, BAX, EGFR, E-Cadherin, N-Cadherin, CCNB1, CDK1 were involved in one signaling network.

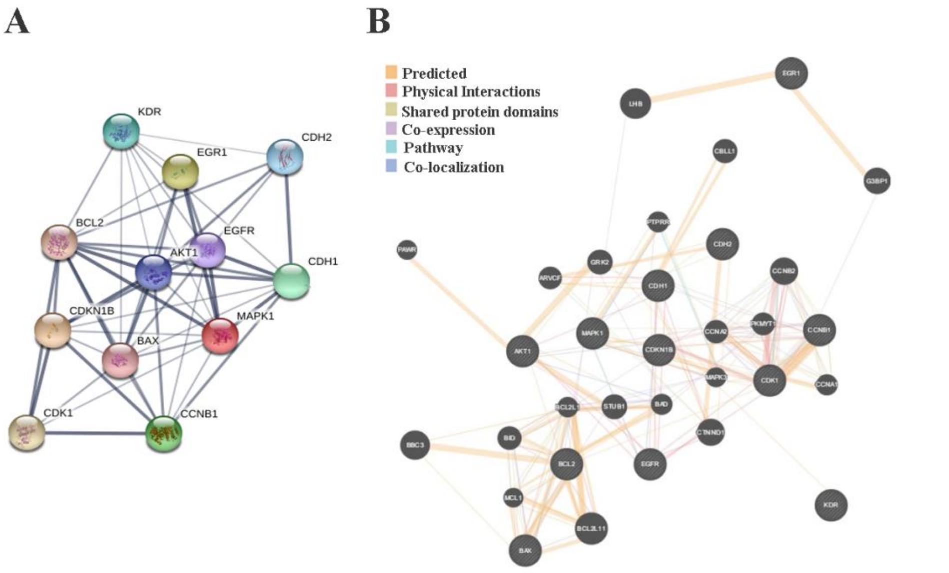

Supplement: Supplementary file 1 — Supplementary information [file 41598_2018_20859_MOESM1_ESM.pdf]
